# The human, F-actin-based cytoskeleton as a mutagen sensor

**DOI:** 10.1186/s12935-017-0488-5

**Published:** 2017-12-12

**Authors:** Nicolette M. Clark, Carlos A. Garcia Galindo, Vandan K. Patel, Michele L. Parry, Rebecca J. Stoll, John M. Yavorski, Elizabeth P. Pinkason, Edna M. Johnson, Chelsea M. Walker, Joseph Johnson, Wade J. Sexton, Domenico Coppola, George Blanck

**Affiliations:** 1 Department of Molecular Medicine, Morsani College of Medicine, University of South Florida, Bruce B. Downs Bd., Tampa, FL 12901 USA; 2 Analytical Microscopy Core Facility, Moffitt Cancer Center and Research Institute, Tampa, FL USA; 3 Department of Genitourinary Oncology, Moffitt Cancer Center and Research Institute, Tampa, FL USA; 4 Department of Pathology, Moffitt Cancer Center and Research Institute, Tampa, FL USA; 5 Immunology Program, Moffitt Cancer Center and Research Institute, Tampa, FL USA

**Keywords:** Mutation frequency, Cytoskeleton, Chemotherapy sequelae, Smoking mutations

## Abstract

**Background:**

Forty years ago the actin cytoskeleton was determined to be disrupted in fibroblasts from persons with DNA repair-defective, hereditary colon cancer, with no clear connection between the cytoskeleton and DNA repair defects at that time. Recently, the large number of sequenced genomes has indicated that mammalian mutagenesis has a large stochastic component. As a result, large coding regions are large mutagen targets. Cytoskeletal protein-related coding regions (CPCRs), including extra-cellular matrix proteins, are among the largest coding regions in the genome and are indeed very commonly mutated in cancer.

**Methods:**

To determine whether mutagen sensitivity of the actin cytoskeleton could be assessed experimentally, we treated tissue culture cells with 4-(methylnitrosamino)-1-(3-pyridyl)-1-butanone and quantified overall cytoskeleton integrity with rhodamine-phalloidin stains for F-actin.

**Results:**

The above approach indicated cytoskeletal degradation with increasing mutagen exposure, consistent with increased mutagenesis of CPCRs in TCGA, smoker samples, where overall mutation rates correlate with CPCR mutation rates (R^2^ = 0.8694; p < 0.00001). In addition, mutagen exposure correlated with a decreasing cell perimeter to area ratio, raising questions about potential decreasing, intracellular diffusion and concentrations of chemotherapy drugs, with increasing mutagenesis and decreasing cytoskeleton integrity.

**Conclusion:**

Determination of cytoskeletal integrity may provide the opportunity to assess mutation burdens in nonclonal cell populations, such as in intact tissues, where DNA sequencing for heterogeneous mutation burdens can be challenging.

**Electronic supplementary material:**

The online version of this article (10.1186/s12935-017-0488-5) contains supplementary material, which is available to authorized users.

## Background

Cytoskeleton protein-related coding regions (CPCRs) are among the most commonly mutated coding regions in the cancer genome atlas, consistent with the large stochastic component to mutagenesis and their large coding regions sizes [1]. In addition, various avenues of research have indicated that mutant CPCRs are likely to impact cancer development [2–5]. Regulatory anomalies related to cancer development have also implicated the cytoskeleton, although over the long history of this topic, there have been contradictory implications of the impact of the disorganized cytoskeleton on cancer progression [6–10]. And, it is clear numerous other conditions and medical issues are related to CPCR mutations [11, 12], potentially including heart and lung damage from mutagenic, cancer chemotherapy [13]. Yet, there has never been a direct test of the sensitivity of the cytoskeleton to mutagen exposure, in an experimental setting. In this report, we have examined the impact of in vitro mutagen treatments on the cytoskeleton, particularly in normal skin fibroblasts, with results indicating that cytoskeleton may provide an opportunity to assess mutation burdens in a highly cost-efficient manner, albeit with little specificity. Such an opportunity could be useful in settings where frequent (but cost-effective) monitoring of mutation burdens could reduce negative health consequences, such as the monitoring of children receiving chemotherapy.

## Materials and methods

### Melanoma cells, exome sequencing, phalloidin staining, and microscopy

WM9 melanoma cells [14] were maintained in a CO_2_-incubator, in tissue culture, with RPMI, penicillin, streptomycin, glutamine, pyruvate and 10% fetal bovine serum; treated with 4-(methylnitrosamino)-1-(3-pyridyl)-1-butanone (NNK; Fluka), as follows. Ten milligrams (mg) of NNK were dissolved into DMSO to generate a 2 mg/ml (1000×) stock. A 30% confluent flask of WM9 cells were treated with 2 μg/ml of NNK, following use of NNK for tissue culture as described in Ref. [15]. After 4 days, cells were trypsinized and re-plated at low dilution for isolating individual colonies. The colonies were amplified and whole-exome sequencing was performed by the Functional Genomics Core Facility at the Moffitt Cancer Center and Research Institute (Tampa, FL). Briefly, one microgram of genomic DNA recovered from the NNK treated, WM9 subclones was used to generate a sequencing library using the Kapa Library Preparation Kit (Kapa Biosystems, Inc., Wilmington, MA). The size and quality of the library was evaluated using the Agilent BioAnalzyer, and equimolar amounts were used for a whole-exome enrichment using the Roche NimbleGen SeqCap EZ Exome Library v2.0 kit, which targets 64 Mb of genomic DNA sequence (Roche NimbleGen, Inc., Madison WI). Quantitative PCR was then used to quantitate the library with the Kapa Library Quantification Kit. Each enriched DNA library was sequenced on the Illumina NextSeq500 sequencer to generate 75–80 million, 75-base paired-end reads for a final average target coverage depth of about 100×. The raw sequence data were processed for alignment and variant calling using the BWA-Enrichment v2.10 software on the Illumina BaseSpace web application, which utilizes the BWA aligner and the GATK framework [16–18]. The verification of the WM9 cell line was obtained with the sequencing results representing the BRAF V600E mutation (rs113488022) as well as via previous study [14, 19]. For rhodamine-phalloidin staining, microscopy and quantification of rhodamine-phalloidin binding, the WM9 subclones were thawed in 10% FBS in glass bottom, 20 mm diameter micro-wells with #1 cover glass (0.13–0.0.16 mm) (In Vitro Scientific). The media was replaced the next day with 0.1% FBS after washing the cells twice with serum free media. After 48 h, cells were fixed with a 1 ml solution containing 50 µg/ml lysopalmitoylphosphatidylcholine in 3.7% formaldehyde. Ten units/ml of rhodamine-phalloidin (Biotium) were added immediately after fixation and cells were placed for 20 min at 4 °C, and washed twice with PBS for microscopy.

### NNK treatment of foreskin fibroblasts

Pre-crises diploid fibroblasts were grown as described above for WM9 cells, plated at 10% confluence in 35 mm plates, and treated the following day with NNK in twofold serial dilutions, beginning with 4 μg/ml, twice the NNK concentration used for isolation of the WM9 subclones above, for a total of eleven NNK concentrations and one untreated sample. The process was duplicated to have one set of treatments for measuring cell viability and one set of treatments for cell expansion, storage and analysis. The cell expansion process was strictly limited prior to freezing to minimize overgrowth of any one variant until the rhodamine-phalloidin analysis was completed. Seven days post-NNK treatment, the cells were transferred to 75 cm^2^ flasks, and 7 days after that first transfer, four vials of the NNK treated fibroblasts were frozen. For phalloidin staining, microscopy and quantification of phalloidin binding, the fibroblasts were thawed in 10% FBS. The media was replaced the next day with 0.1% FBS after washing the cells twice with serum free media. The cells were cultured for an additional 48 h, fixed, and stained with rhodamine-phalloidin, as indicated above for the WM9 melanoma subclones. Microscopy is described below.

### Raji B cell phalloidin assay

A saturated culture of Raji B-cells (2 million cells/ml) was diluted 1/6 and treated with NNK concentrations as indicated in Fig. 5. Cells were re-diluted 1/6 3 days after NNK treatment, diluted again 1/4 and treated with 2 μM Entinostat, which leads to apoptosis and perforation of the membranes for subsequent rhodamine-phalloidin and DAPI treatment prior to flow cytometry. After 48 h, cells are pelleted, re-suspended in PBS with 2% human serum, to which was added 1 μg/ml DAPI and 10 units/ml rhodamine-phalloidin. The cells were left at 4 °C for 20 min and then assayed by flow cytometry for rhodamine and DAPI absorbance.

### Microscopic analyses

Cells in glass bottom wells were observed with a Zeiss Z1 Observer inverted microscope through a 20×/0.5NA Plan Apo objective (Carl Zeiss GmbH, Germany). An EXFO X-CITE 120 light source (Lumen Dynamics Group Inc, Mississauga, Canada) and filter cube (Chroma Technology Corporation, Vermont, USA) were used for viewing. Fluorescence and phase contrast images were captured with an AxioCam MRm3 CCD camera and Axiovision 4.8.2 software (Carl Zeiss GmbH, Germany).

### ImageJ quantification

For the WM9 melanoma subclones, the ImageJ, Cell-Counter Analyzing tool was used to count each cell in the overlapping phase and rhodamine images; the cell counts were tabulated under “Results”. The Measure tool was used to obtain the mean value for brightness, per the user manual (http://imagej.nih.gov/ij/docs/guide/user-guide.pdf). The mean value for brightness was then divided by the number of cells in each image. For the fibroblasts, five images were obtained for each of the 12 wells with different concentrations of NNK treated fibroblasts. From each of these five images, five individual cells were exported as individual images. Each cell was selected on the basis of having a clear boundary, having no other overlying cells, and being completely included in the image. The individual cells were then exported as individual images, with a black background. Thus a total of three hundred images of individual cells were obtained. Each image was opened in ImageJ and the brightness and contrast controls were open. Auto button for Brightness and Contrast controls was selected to automatically optimize brightness and contrast based on an analysis of the image’s histogram. When selected, the entire image was optimized based on the ImageJ analysis of the selection. The ImageJ optimization was done by allowing a small percentage of pixels in the image to become saturated. To quantify the red color of the rhodamine-phalloidin, we selected Analyze, then Histogram, then RGB to obtain an optimized histogram. This histogram was termed “Red Histogram of [filename]” by the ImageJ software. The histogram values were then imported into Excel and the average was calculated. The average was then divided by the area of each cell. The ImageJ version 1.48v was used.

### Analysis of the cancer genome atlas (TCGA) lung adenocarcinoma (LUAD) mutations from smokers

The TCGA (http://cancergenome.nih.gov/) smoker LUAD data was accessed as described [20]. Briefly, barcodes representing current smokers and total mutations were downloaded, and the Excel COUNTIF macro was used to determine the number of mutations per barcode for each of the current smoker barcodes. The mutation data for the HUGO symbols for the CPCR set were copied to a second Excel sheet and the COUNTIF macro was used to obtain CPCR mutations per barcode. The data was access with approval via National Institutes of Health Data Access Committee Request #27073-3 for Project #6300.

### Exome sequences of Moffitt Cancer Center, bladder cancer patients

The exome sequences for the bladder cancer specimens were obtained exactly as described [21], using the Moffitt Cancer Center functional genomics core facility, USF IRB (Ethics) Approval Number Pro00022538 (Additional files 1, 2, 3 and 4).

### Quantification of anti-F-actin staining of the bladder cancer samples

Formalin fixed, paraffin embedded (FFPE) bladder cancer samples (USF IRB Approval Number Pro00022536, July 16, 2015) were stained with a polyclonal anti-F-actin (Bioss, Catalog Number bs1571R) antibody at the Moffitt Cancer Center histology core facility. Image J (version 1.48v) was used to quantify F-actin binding. Images were opened into the ImageJ software. Using the brightness and contrast menu (image, adjust, brightness and contrast) the auto feature was selected. ImageJ was used to create a histogram of each cell (analyze, histogram—shortcut CRTL+H), per the Image J’s user guide: “With RGB images, the default histogram is calculated by converting each pixel to grayscale using the formula gray  =  (red  +  green  +  blue) ⁄ 3 or gray  =  0.299  × red  +  0.587  × green  +  0.114  × blue” (https://imagej.nih.gov/ij/docs/guide/146-30.html#toc-Subsection-30.10). The grey-scale histograms were then copied into Excel for further analysis (see Additional file 5). Using images that contain large amounts of F-actin staining (brown), an area was selected that specifically represented F-actin binding. From this selection a plot profile was created representing the measurement of F-acting binding in each cell (see Additional files 5, 6).

### Second harmonic generation (SHG) microscopy and image quantification of melanoma cell lines

Second harmonic generation [22, 23] images were captured through a 25×/0.95NA water objective lens with a Leica SP5 Multiphoton Microscope (Leica Microsystem GmbH, Wetzlar, Germany) equipped with a MaiTai DeepSee Ti–sapphire laser (Spectra-Physics Inc., Mountain View, CA) and HyD detectors. The MP laser was tuned to 880 nm and emissions were collected through a 440 nm band pass filter in order to achieve SHG imaging. In addition to SHG, bright field images of the identical fields were captured an Argon laser tune to 488 nm and transmitted light PMT. All images and overlays were prepared in Leica LASAF software (Leica Microsystems GmbH, Wetzlar, Germany). SHG signal from each image was evaluated using Definiens Developer version 2.4 (Definiens AG, Munich, Germany). Within this software an auto-threshold algorithm was used to segment the SHG signal from the background and determine total fluorescence signal from SHG in each image. Finally SHG signal per cell was calculated by using the bright field image to enumerate the total number of cells in each SHG image. Further details are in Additional file 7.

## Results

**Fig. 1:**
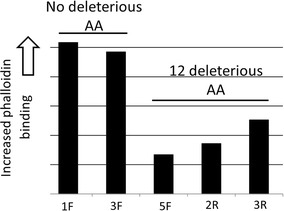
Quantification of rhodamine-phalloidin binding and summation of CPCR deleterious amino acids in NNK treated, WM9 subclones. Quantification of phalloidin binding is described in “Materials and methods”. See also Table 1 and Additional file 8. The p value for comparison of deleterious amino acids, for the high and low phalloidin-binding subclones is < 0.0004. However, there is no significant difference between the two types of subclones in terms of total unique and shared CPCR mutations, i.e., mutations shared with another subclone in the same high or low phalloidin-binding group. Thus, the high phalloidin-binding subclones had a total of 357 unique and shared mutations and the low phalloidin-binding subclones had a total of 569 mutations, in the CPCR set. The HUGO symbols for the CPCR set, for both Figs. 1 and 6, were obtained from Ref. [25] are: ANK2, APC, COL11A1, DNAH10, DNAH11, DNAH3, DNAH5, DNAH7, DNAH8, DSCAM, DST, FAT3, FAT4, FBN2, FGFR1, FLG, MUC16, MUC17, MUC4, NEB, NEFH, NF1, PCDH15, PCDHAC2, PCDHGC5, PCLO, PKHD1, PLEC, RELN, SPTA1, SPTAN1, SSPO, SYNE1, SYNE2, TTN, and XIRP2

We treated WM9 melanoma cells with NNK [15] and isolated five clones for phalloidin binding, to assess the integrity of the F-actin-based cytoskeleton [7] (Fig. 1) and for exome sequencing. The SNVs unique to each clone were identified (Additional file 8) and analyzed with the web based PROVEAN tool (PROVEAN v1.1.3, using a cutoff value of − 2.5, with “more negative” representing more deleterious; http://provean.jcvi.org/). Results indicated that the three subclones with the lowest level of rhodamine-phalloidin staining also had a large number of deleterious mutations among a CPCR set, previously defined by the frequency of mutated CPCRs in five cancer datasets [20]. The two subclones with the higher level of rhodamine-phalloidin staining had no deleterious mutations (Fig. 1; Table 1; p < 0.0004, Students t test). These results suggested that rhodamine-phalloidin binding could represent a measure of cytoskeleton integrity following mutagen exposure and prompted consideration of a different experimental design, which allowed for a more comprehensive opportunity for determination of statistical significance; and allowed for an opportunity to assess mutagen impact in a nonclonal cell population.

**Table 1:** Coding regions representing deleterious amino acid mutations in NNK-treated, low phalloidin-binding, WM9 subclones

HUGO symbol of CPCR coding region	WM9-5F	WM9-2R	WM9-3R
DNAH11		1 unique	1 unique
MUC16	1 unique *1 shared with 3R*	1 unique	1 unique *1 shared with 5F*
TTN			1 unique
XIRP2	*2 shared with 2R*	*2 shared with 5F*	

No deleterious amino acid alterations were detected in the high phalloidin binding, WM9-1F and WM9-3F subclones; shared mutations indicated in italics. The three low phalloidin binding subclones (WM9-3R -2R, and -5F) represent a total of 12 deleterious amino acid changes, based on the PROVEAN tool (see Additional file 8)

**Fig. 2:**
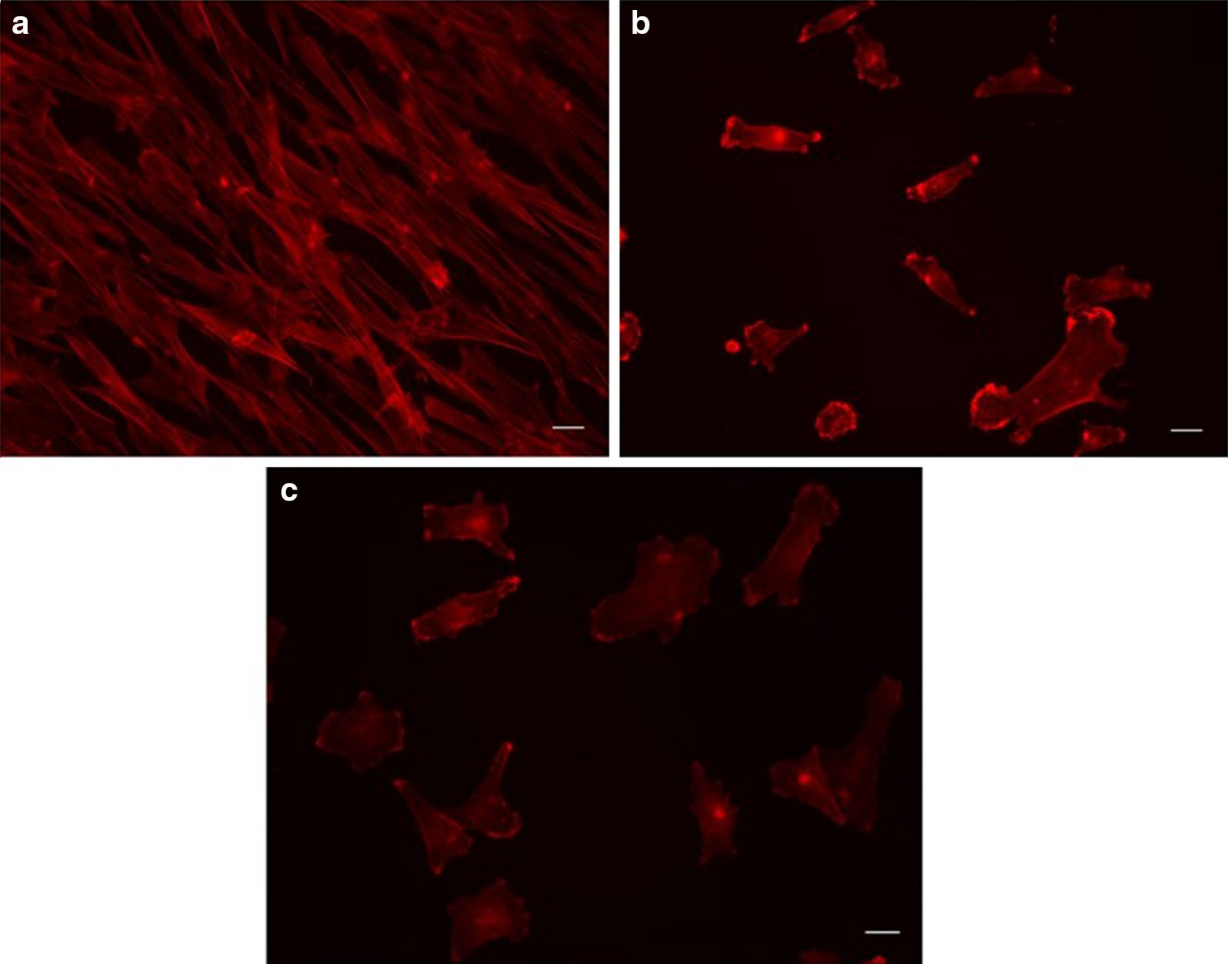
Contrasting rhodamine-phalloidin stains of a WM9 melanoma subclone and foreskin fibroblasts. **a** Rhodamine-phalloidin stain of human, pre-crises foreskin fibroblasts used in Fig. 3. **b** Rhodamine-phalloidin stain of the subclone WM9-1F (a low mutation WM9 subclone). **c** WM9-3R, a high mutation, WM-9 subclone (Fig. 1; Table 1) shown here for comparison with the WM9-1F image (**b**). Scale bar, bottom right in all images, is 10 μm

**Fig. 3:**
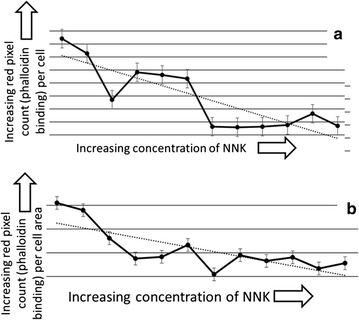
The impact of the exposure to NNK on foreskin fibroblasts, rhodamine-phalloidin binding. **a** Correlation of mutagen exposure and overall red pixel count (rhodamine-phalloidin binding), per cell. R^2^ = 0.7177; p < 0.004. **b** Correlation of mutagen exposure and red pixel count (rhodamine-phalloidin binding), per cell-area. R^2^ = 0.5983; p < 0.004. For additional detail see “Materials and methods” and Additional file 9

The rhodamine-phalloidin detected cytoskeleton in pre-crises, foreskin fibroblasts (Fig. 2a) had more extensive structure than the phalloidin-detected cytoskeleton in the melanoma cells (Fig. 2b), not surprising, considering the association of disrupted cytoskeleton with tumorigenesis [this more extensive structure is in evidence when comparing the foreskin fibroblasts to either the low or high mutation WM9 subclones (Fig. 2a–c)]. The additional structure offered the opportunity of mutagen-sensitivity over a wider range. To meet the above goals, we treated the fibroblasts with eleven, twofold serial dilutions of NNK, allowed for several rounds of division, stained the fibroblasts with rhodamine-phalloidin, and quantified the rhodamine-phalloidin binding using the ImageJ software. Results indicated a statistically significant correlation in the reduction of rhodamine-phalloidin staining with increasing levels of mutagen exposure, whether calculated as rhodamine-phalloidin binding per cell (Fig. 3a; R^2^ = 0.7177; p < 0.004) or per unit area (of the cells) (Fig. 3b; R^2^ = 0.5983; p < 0.004).

**Fig. 4:**
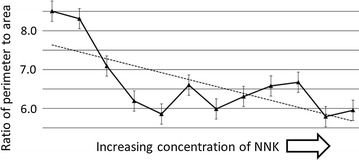
Correlation of mutagen exposure and the decrease in the cell perimeter to area ratio. R^2^ = 0.5043; p < 0.01. For additional detail see “Materials and methods” and Additional file 10

We considered the possibility that the mutagen impact on the cytoskeleton could also impact cell shape. Thus, we obtained the perimeters and areas for 25 cells for each mutagen treatment level, and for the untreated cells, with results indicating a downward progression in the perimeter to area ratios (Fig. 4; R^2^ = 0.5043; p < 0.01).

**Fig. 5:**
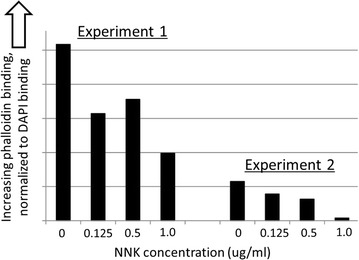
Raji B-cell, rhodamine-phalloidin binding following exposure to NNK. See Additional file 11

To determine whether the impact of mutagen on rhodamine-phalloidin binding could be detected for non-adherent cells, we treated Raji B-cells with NNK, or mock-treated the cells, and assessed rhodamine-phallodin binding by flow cytometry, with results indicating reduced phalloidin binding after the NNK exposure (Fig. 5).

**Fig. 6:**
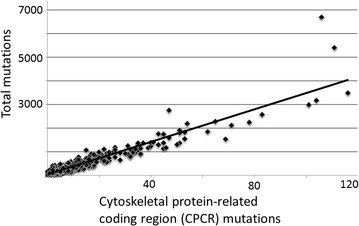
Correlation of TCGA LUAD smoker mutations and CPCR mutations. The mutation counts were obtained as indicated in “Materials and methods”, with further detail in Additional file 12. R^2^ = 0.8694; p < 0.00001. The CPCRs included in the above analysis is identical to Ref. [20] and is included in the legend to Fig. 1

To obtain a better understanding of the CPCR set as a mutagen sentinel set in vivo, we assessed the mutation occurrences in this set, as well as total number of mutations, in smokers among the LUAD (lung adenocarcinoma) dataset in TCGA. Results indicated that the CPCR mutations correlate with overall mutation counts, with a high degree of statistical significance (Fig. 6; R^2^ = 0.8694; p < 0.00001).

**Fig. 7:**
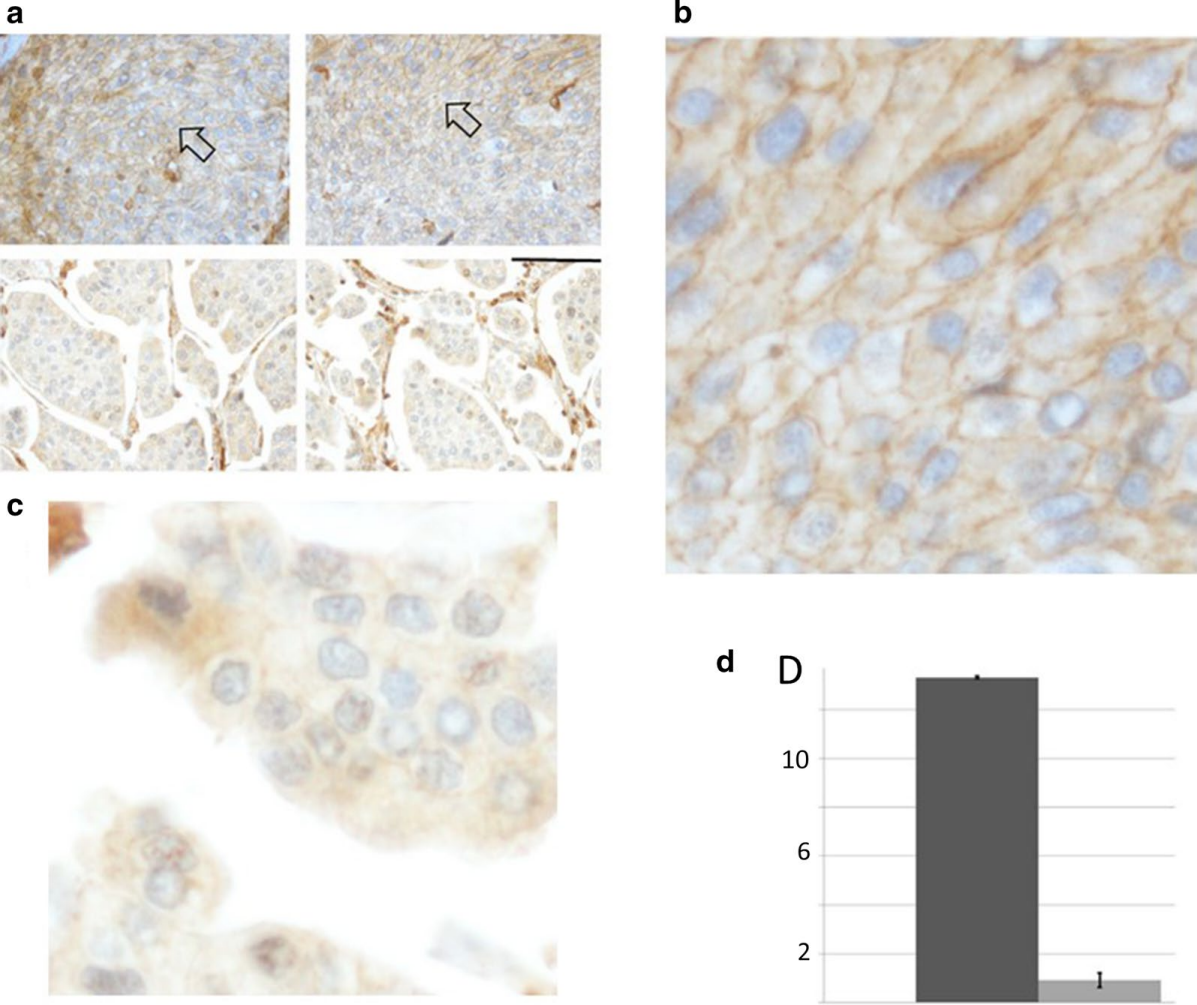
Anti-F-actin stains of MCC-BLCA1 and MCCBLCA-2. **a** FFPE, anti-F-actin stains of MCC-BLCA1 (top 2 panels; low mutation rates) and MCC-BLCA2 (bottom 2 panels; high mutation rates). **b** Higher magnification of arrowhead region of upper right panel of **a**, to show more precise image of anti-F-actin stain. **c** Higher magnification of the lower right panel of **a**, center of image of **a** lower right panel, emphasizing the reduced level of anti-F-actin staining. **d** Quantification of anti-F-actin binding to the bladder cancer cells (BLCA1, left side; BLCA2, right side). In **a**, the anti-F-actin binding to the cells is represented by the brown lines at the edges of the cells, indicated by arrowheads. See Additional files 5 and 6, particularly for explanation of **c** quantification units on y-axis

To determine whether the above indicated correlation of a high rate of mutagenesis and the degradation or loss of F-actin-based, cytoskeletal structures could be observed in a patient setting, we obtained the exome sequence of 16 Moffitt Cancer Center bladder cancer patients. After removal of SNPs, the number of remaining single nucleotide variants (SNVs) ranged from 1962 to 5054, with the majority of the samples very close to the lower number of SNVs. These two samples represented 53 (BLCA1) and 89 (BLCA-2) mutations in the above indicated CPCR set [20] (Fig. 1), respectively (see Additional files 2, 3, 4). We next stained FFPE tissues representing the two extreme bladder cancer samples, with respect to SNV occurrence, with anti-F-actin (Fig. 7a). Although only two patient samples were evaluated, quantification of the results clearly demonstrated continued consistency of the presence of F-actin structures and lower mutation counts (Fig. 7b).

**Fig. 8:**
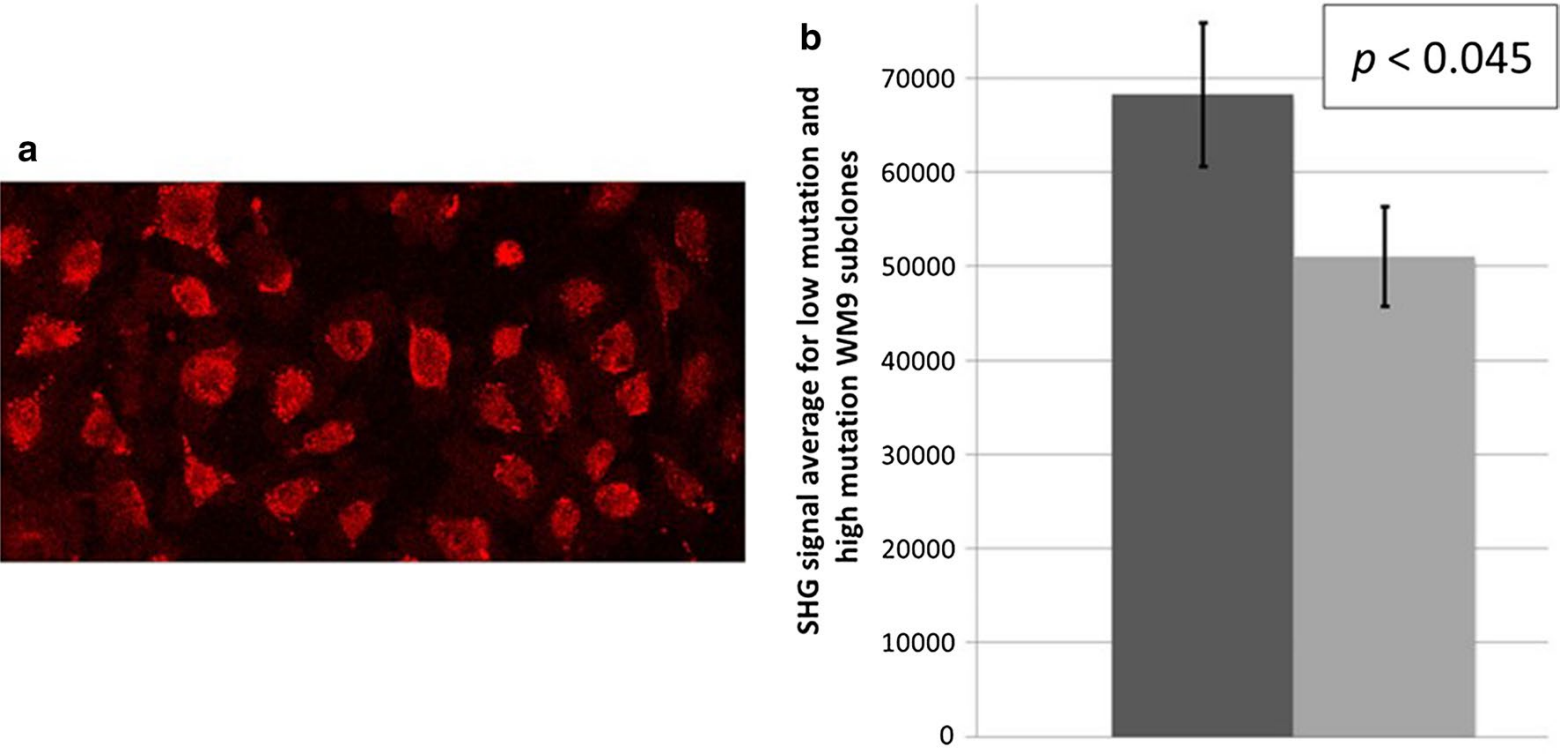
Second harmonic generation (SHG) imaging of the NNK treated, WM9 subclones. **a** Example SHG image of the low mutation subclone, WM9-3F. **b** Quantification of the SHG signal (low mutation subclones, left bar; high mutation subclones, right bar) (see Additional file 7)

Finally, to identify a second approach, besides the F-actin dependent approach, for assessing the impact of mutations on ordered structure arising from large polypeptide subunits forming polymers, we obtained SHG images of the NNK exposed WM9 subclones with fewer or more mutations. We obtained images for two of the low mutation subclones, WM9-1F and -3F; and two high mutation subclones, WM9-5F and 3R (Fig. 8a). Quantification of the results (Fig. 8b) indicated a 25% decrease in pixel intensity representing ordered polymers in the higher mutation WM9 subclones, with *p* < 0.04 (Additional file 7).

## Discussion

Taken together, the above results are consistent with the conclusion that rhodamine-phalloidin binding reflects mutant cytoskeletal proteins that reduce the integrity of the F-actin based cytoskeleton. This type of “integrity reduction” is likely traceable to the fact that the cellular cytoskeleton is a polymeric structure composed of many subunits representing at least several, if not many distinct proteins. Thus, a large number of coding regions may be susceptible to mutation such that any one of the resultant mutant, cytoskeletal related proteins could corrupt the polymer, akin to what is seen in osteogenesis imperfecta, whereby mutant collagen subunits interfere with polymer and cartilage formation in a dominant negative fashion [11]. As an alternative possibility, it is conceivable that more mutagen would make it more likely that a point mutation could affect a regulatory pathway, in turn impacting the cytoskeleton [9, 10]. However, it is highly improbable that such rare mutagenic events would lead to a significant and strong correlation coefficient representing the mutagen increases and the impact on the phalloidin binding (Figs. 3, 5) and on the perimeter to area ratios (Fig. 4).

In 1977, Pollack and colleagues observed that the actin-cytoskeleton was disrupted in fibroblasts from persons with DNA repair-defective, hereditary colon cancer [24]. Given the above results and the points in the preceding paragraph, the most likely explanation for that observation is that the DNA repair defects led to mutations in CPCRs, which in turn lead to a disorganized, actin-cytoskeleton (Additional file 12).

Our results also indicate that more mutagen exposure leads to a reduced perimeter to area ratio, which raises questions about the basis for drug resistance in cancer. Many articles have indicated that the result of selecting for resistance to a variety of anticancer drugs is a spheroid cell, often invoking epithelial to mesenchymal transition as a process fundamental to both cell shape change and drug resistance. However, it is clear that the CPCR set is a large mutagen target and loss of cytoskeleton integrity may lead to reduced cell spreading and thus a reduced surface area to volume ratio, in our results represented as a reduced perimeter to area ratio. With such a shape alteration, reduced intra-cellular drug concentrations, and reduced intra-cellular drug diffusion should be considered as possible explanations for why drug resistance selects for spherical cells. In fact, given the vulnerability of the CPCR set to mutation, mutagenic drug treatments, common cancer therapies, may both create and select for drug resistance.

Finally, it is important to note the likely opportunity to take advantage of SHG microscopy for obtaining crude but potentially medically useful indications of mutation burdens in persons. For example, in some cases smoking may lead to an accumulation of mutation burdens easily assessable via a buccal swab and SHG microscopy. This type of approach may conveniently and inexpensively distinguish between persons who have suffered numerous mutations and those with low mutation burdens. A similar monitoring possibility could be useful for children treated with anti-cancer chemotherapy. An SHG based monitoring strategy would be far less expensive and less logistically complicated than DNA sequencing and thereby allow for very frequent, long-term monitoring. In particular, assessing mutation burdens via the impact of mutations on cytoskeletal or extra-cellular matrix structures would eliminate the need for a clonal population of cells for DNA sequencing or the need to do single cell sequencing. That is, assaying the mutation burdens via loss of subcellular structures need not require that mutations be in the same gene or at the same nucleotide position. This type of assay approach would allow for using a heterogeneous set of mutant cells as the basis of a mutation burden assessment.

## Conclusion

The above work supports the idea that an indication of mutation burdens can be had by assessing the actin-cytoskeleton integrity, consistent with the idea that large coding regions are, based on probability, frequent mutagen targets and can thereby often express dominant negative mutant polypeptides that corrupt polymer formations. These results may lead to cost-effective approaches for monitoring mutation burdens where a high level monitoring frequency, or other circumstances, prevent relatively costly DNA sequencing approaches, for example for monitoring the impact of mutagenic chemotherapy.

## Additional files

**Additional file 1.** IRB approval document.

**Additional file 2.** BLCA1 mut counts.

**Additional file 3.** BLCA2 mut counts.

**Additional file 4.** BLCA CPCR mut counts.

**Additional file 5.** F-actin quantification.

**Additional file 6.** F-actin IHC quantification method.

**Additional file 7.** SHG quantification.

**Additional file 8.** WM9 subclones.

**Additional file 9.** NNK fibroblasts.

**Additional file 10.** Perimeter area ratios.

**Additional file 11.** Smoking LUAD.

**Additional file 12.** Raji B-cell phalloidin.

## Abbreviations

CPCR: cytoskeletal protein-related coding region
ECM: extra-cellular matrix
NNK: 4-(methylnitrosamino)-1-(3-pyridyl)-1-butanone
TCGA: the cancer genome atlas
SNV: single nucleotide variant
LUAD: lung adenocarcinoma
HUGO: human genome organization
