# Supplementary material for: The human, F-actin-based cytoskeleton as a mutagen sensor
Source: Cancer Cell Int. 2017 Dec 12;17:121. doi: 10.1186/s12935-017-0488-5 (PMC5727871; doi:10.1186/s12935-017-0488-5)

**Garcia-Galindo et al SOM 2016, F-action IHC quantification method**

The images below were taken from the Moffitt Cancer Center, bladder tumor samples indicated in the accompanying article, MCC-BLCA1 and MCC-BLCA2. The images represent immunohistochemistry stains for F-actin. To verify and refine the use of the 0-255 grey scale for quantification, five images (yellow squares below) were obtained that represented the F-actin stain only. As can be seen by inspection of the Y-axes, these images define the scale 90-150 range used for quantification in the accompanying SOM Excel file labeled, “Garcia Galindo et al SOM 2016, F-actin quantification”. The pixel counts were then used for the Fig. 7B display item (bar graph) in the article.


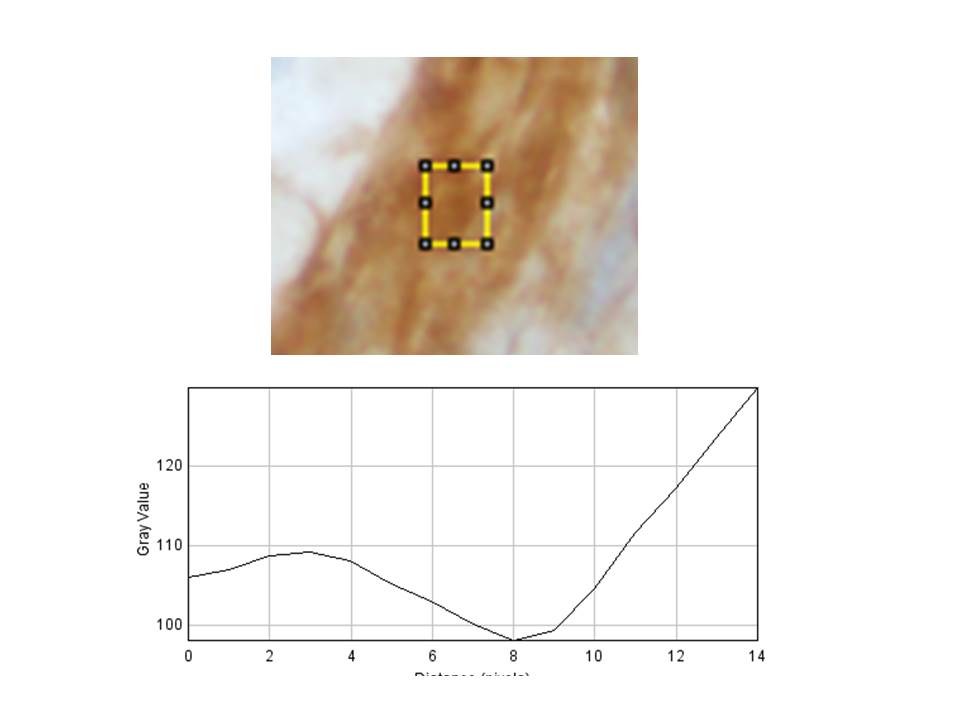

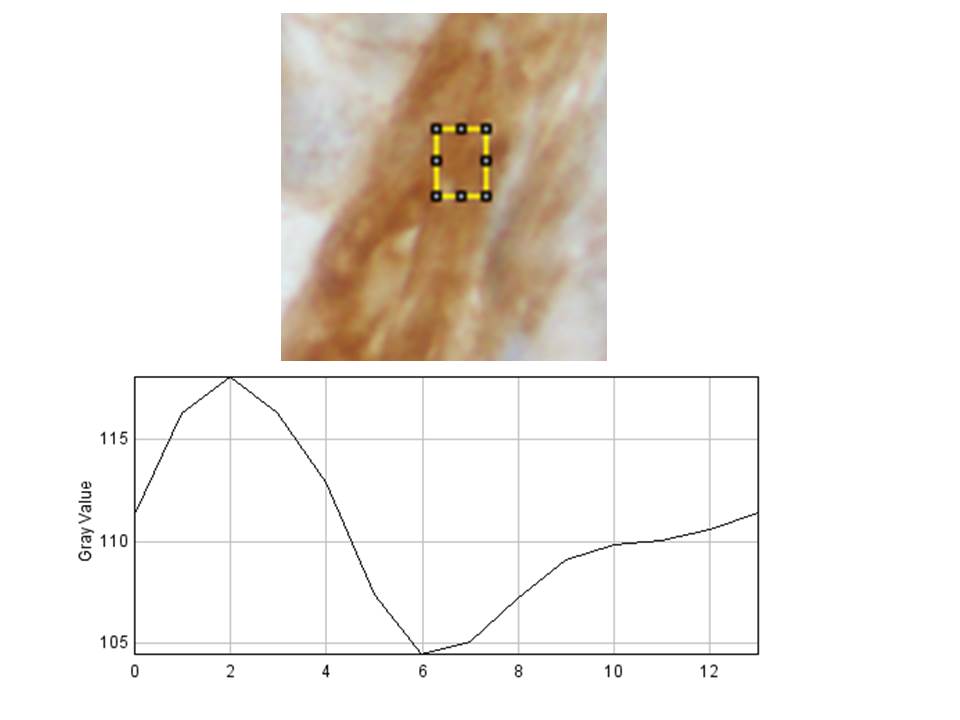

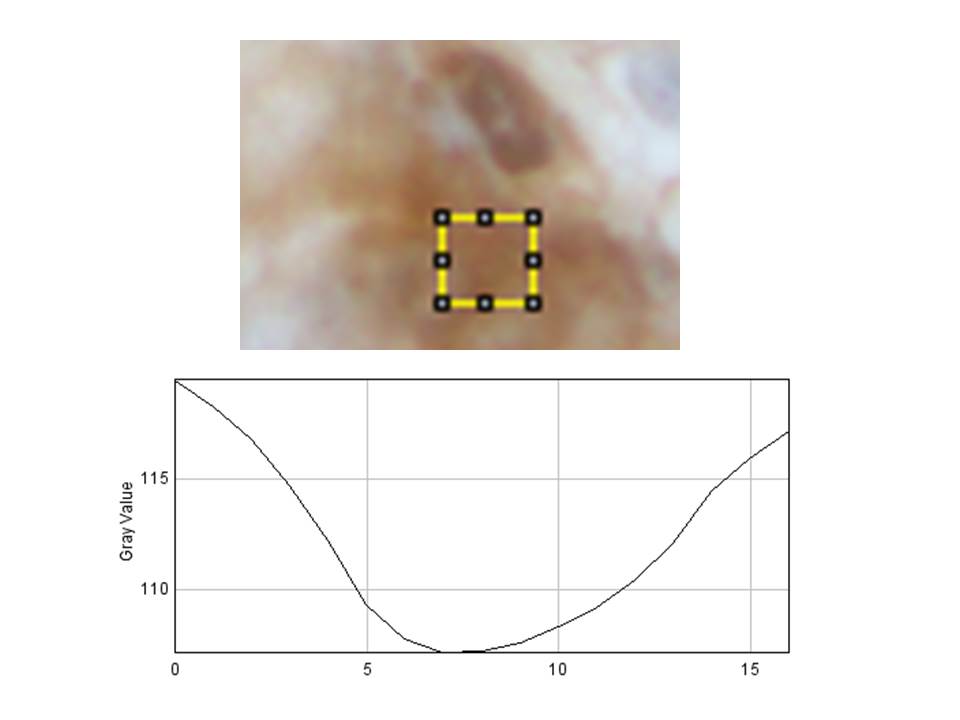

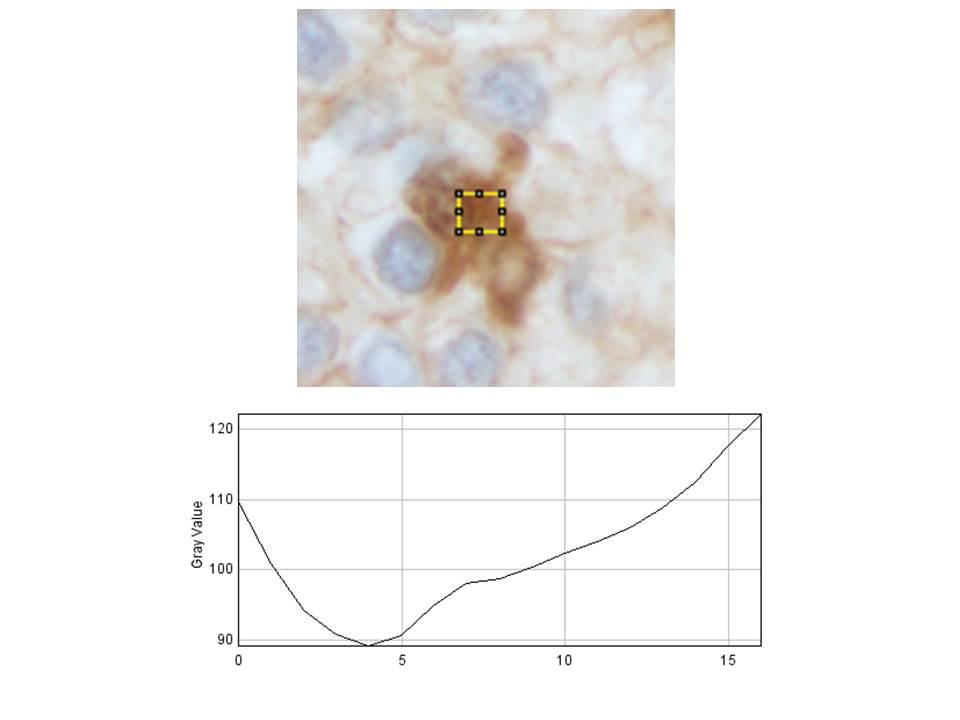

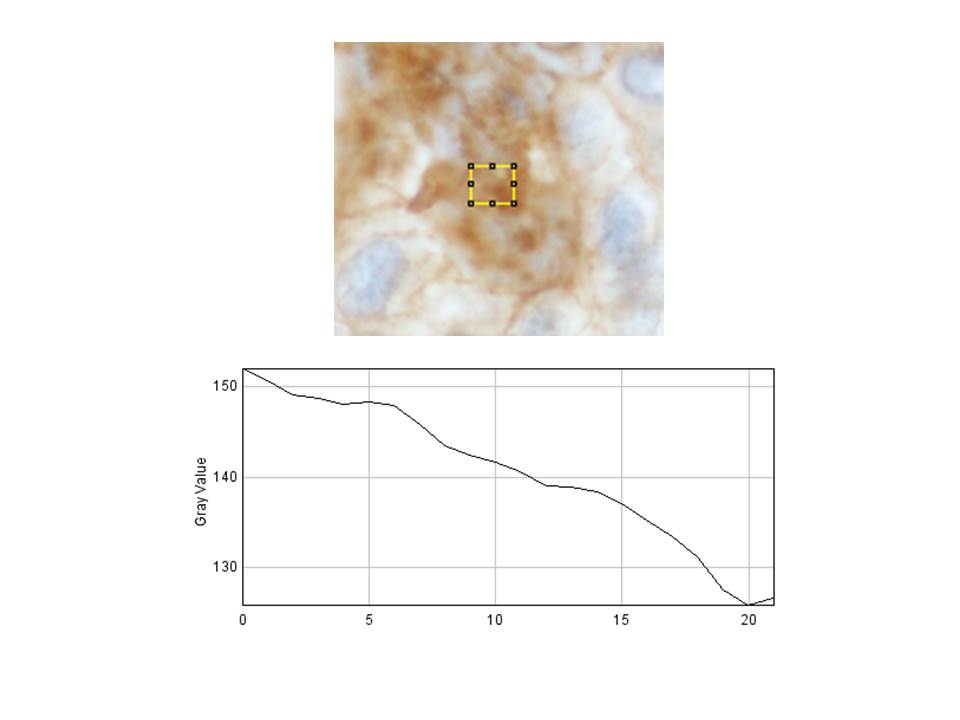

Supplement: Supplementary file 6 — Additional file 6. F-actin IHC quantification method. [file 12935_2017_488_MOESM6_ESM.docx]
