# Supplementary material for: The human, F-actin-based cytoskeleton as a mutagen sensor
Source: Cancer Cell Int. 2017 Dec 12;17:121. doi: 10.1186/s12935-017-0488-5 (PMC5727871; doi:10.1186/s12935-017-0488-5)
Supplement: Supplementary file 1 — Additional file 1. IRB approval document. [file 12935_2017_488_MOESM1_ESM.pdf]

USF UNIVERSITY OF SOUTH FLORIDA

RESEARCH INTEGRITY AND COMPLIANCE
Institutional Review Boards, FWA No. 00001669
12901 Bruce B. Downs Blvd., MDC035 • Tampa, FL 33612-4799
(813) 974-5638 • FAX (813) 974-7091

7/16/2015

George Blanck, Ph.D.
Molecular Medicine
12901 Bruce B. Downs Bd.
MDC 7
Tampa, FL   33612

RE: **Expedited Approval for Initial Review**
IRB#: Pro00022538
Title: Cytoskeletal protein related coding region mutations in bladder cancer.

Study Approval Period: 7/16/2015 to 7/16/2016

Dear Dr. Blanck:

On 7/16/2015, the Institutional Review Board (IRB) reviewed and **APPROVED** the above application and all documents contained within, including those outlined below.

Approved Item(s):
Protocol Document(s):
same, no highlighting of revisions

It was the determination of the IRB that your study qualified for expedited review which includes activities that (1) present no more than minimal risk to human subjects, and (2) involve only procedures listed in one or more of the categories outlined below. The IRB may review research through the expedited review procedure authorized by 45CFR46.110 and 21 CFR 56.110. The research proposed in this study is categorized under the following expedited review category:

(5) Research involving materials (data, documents, records, or specimens) that have been collected, or will be collected solely for nonresearch purposes (such as medical treatment or diagnosis).

Your study qualifies for a waiver of the requirements for the informed consent process as

outlined in the federal regulations at 45CFR46.116 (d) which states that an IRB may approve a consent procedure which does not include, or which alters, some or all of the elements of informed consent, or waive the requirements to obtain informed consent provided the IRB finds and documents that (1) the research involves no more than minimal risk to the subjects; (2) the waiver or alteration will not adversely affect the rights and welfare of the subjects; (3) the research could not practicably be carried out without the waiver or alteration; and (4) whenever appropriate, the subjects will be provided with additional pertinent information after participation.

Your study qualifies for a waiver of the requirement for signed authorization as outlined in the HIPAA Privacy Rule regulations at 45CFR164.512(i) which states that an IRB may approve a waiver or alteration of the authorization requirement provided that the following criteria are met (1) the PHI use or disclosure involves no more than a minimal risk to the privacy of individuals; (2) the research could not practicably be conducted without the requested waiver or alteration; and (3) the research could not practicably be conducted without access to and use of the PHI. A waiver of HIPAA Authorization is granted for this retrospective review of patients who underwent a radical cystectomy for bladder urothelial cancer at Moffitt Cancer Center between January 1, 2007 and June 30, 2015 and who consented to participate in Moffitt's IRB-approved Total Cancer Care study. This waiver allows the study team or its honest broker to obtain PHI of these patients from the TCC database and/or the Moffitt electronic medical record (PowerChart).

As the principal investigator of this study, it is your responsibility to conduct this study in accordance with IRB policies and procedures and as approved by the IRB. Any changes to the approved research must be submitted to the IRB for review and approval via an amendment. Additionally, all unanticipated problems must be reported to the USF IRB within five (5) calendar days.

We appreciate your dedication to the ethical conduct of human subject research at the University of South Florida and your continued commitment to human research protections. If you have any questions regarding this matter, please call 813-974-5638.

Sincerely,

VJorgensen MD

E. Verena Jorgensen, M.D., Chairperson
USF Institutional Review Board
